# Guanylate-binding protein 5: a promising biomarker and therapeutic target

**DOI:** 10.1128/iai.00026-25

**Published:** 2025-08-11

**Authors:** Jianliang Lu, Wei Wang

**Affiliations:** 1 Department of Clinical Laboratory, Xiangya Hospital, Central South University 12570, Changsha, Hunan, People's Republic of China; University of Pittsburgh, Pittsburgh, Pennsylvania, USA

**Keywords:** guanylate-binding protein 5, infection, cancer, immune disease, inflammation, therapeutic target

## Abstract

The guanylate-binding protein (GBP) family, a group of interferon-induced GTPases, is pivotal in pathogen defense, inflammation regulation, and tumor immunity. Among them, GBP5 has emerged as a key player due to its distinctive roles in various diseases. However, existing studies reveal significant gaps, particularly regarding its expression, regulatory mechanisms, and functional dynamics across diverse diseases and patient populations, limiting its reliability as a biomarker or therapeutic target. This review provides a comprehensive synthesis of GBP5 functions across infectious diseases, cancer, immune disorders, and inflammation, with dedicated analysis of its context-dependent functional variability in distinct immune landscapes, genetic backgrounds, and disease progression stages. This systematic evaluation provides a critical foundation for future research, highlighting GBP5’s promise as both a biomarker and therapeutic target in precision medicine.

## INTRODUCTION

Guanylate-binding proteins (GBPs) are a group of interferon-induced GTPases that play a pivotal role in cellular autonomous immunity (1). Initially identified in IFN-γ (interferon-γ) -activated mouse cells, these proteins, ranging from 65 to 73 kDa, are integral components of the human innate immune system, where they provide crucial defense against pathogens (2–4). GBPs, alongside other immune-related GTPases such as immunity-related GTPases (IRGs), myxoma resistance proteins, and very large inducible GTPases, belong to the dynamin GTPase superfamily (1). In humans, the GBP gene family consists of seven members, with GBP1–5 being widely expressed, while GBP6 and GBP7 are primarily localized to epithelial surfaces in the lung and intestinal mucosa (5). Although GBP1 has been the most extensively studied due to its broad antiviral and antimicrobial properties, growing research attention has been directed toward GBP2, GBP3, and GBP5 (4, 6). Among these, GBP5 has garnered significant interest because of its distinctive biological functions and clinical potential. Induced primarily by interferons, GBP5 exerts diverse effects within infection, inflammation, and tumor microenvironments, modulating host immune responses to pathogens through various mechanisms (7–9). The functional significance of GBP5 has been highlighted in several disease contexts. In infectious diseases, such as *Mycobacterium tuberculosis* (Mtb) and human immunodeficiency virus (HIV), GBP5 modulates disease progression through various anti-pathogenic pathways (10, 11). In sepsis-associated liver injury, GBP5 activates the NLR family pyrin domain containing 3 (NLRP3) inflammasome, exacerbating inflammation and tissue damage (12). In inflammatory bowel disease (IBD), GBP5 influences intestinal immunity and alters gut microbiota composition, further demonstrating its role in host-microbe interactions (13). In tissue-specific contexts such as pulpitis, GBP5 contributes to the regulation of local inflammatory responses (7). In oncology, GBP5 has been implicated in promoting the proliferation and migration of gastric cancer (GC) cells (9), and modulating immune interactions within the glioma microenvironment, contributing to tumor progression (14). Additionally, GBP5’s immunoregulatory effects in chronic hepatitis B suggest its involvement in virus-associated tumorigenesis (15).

Despite these findings, GBP5’s expression, regulatory mechanisms, and functional roles exhibit significant variability across different diseases and patient populations, influenced by factors such as immune background, genetic variability, and disease stage. These inconsistencies present challenges for its clinical application as a biomarker or therapeutic target. This review provides a systematic overview of GBP5 research, encompassing its roles in infectious diseases, cancer, and immune-mediated disorders. By analyzing GBP5’s mechanistic variability across diverse pathological settings, we aim to assess its potential as a target for personalized therapies. This comprehensive evaluation underscores GBP5’s promise in advancing precision medicine and offers a foundation for future research directions.

## AN OVERVIEW OF GBP5

Human guanylate-binding protein 5 (hGBP5) belongs to the interferon-induced p65 GTPase family and is strongly induced by pro-inflammatory cytokines (16). In humans, the seven GBP genes are clustered on chromosome 5, whereas in mice, 11 GBPs are distributed across two chromosomes (17). Subcellularly, GBPs predominantly localize in the cytoplasm, with GBP5 concentrated in the perinuclear region and co-localized with the Golgi apparatus (18). These proteins are characterized by their ability to bind guanine nucleotides, displaying similar affinities for GTP, GDP, and GMP (19, 20). The expression of hGBP5 is regulated by interleukins and can be markedly upregulated, sometimes by several 100-fold, in response to IFN-γ stimulation (21). While most studies on GBP5 focus on humans and mice, its innate immune functions have also been described in other organisms, including plants, invertebrates, teleosts, pigs, and *Tupaia* (4, 6, 18, 22, 23).

GBPs play a critical role in broad-spectrum innate immune responses against diverse pathogens, primarily through nucleotide binding and catalytically induced oligomerization (24). GBP5, as a member of the large GTPase superfamily, features an N-terminal large GTPase (LG) domain and one or more stalk domains (25). The stalk domain, also referred to as the C-terminal helical domain, includes the intermediate domain (MD) and the GTPase effector domain (GED). Structural studies reveal that GBP5 forms dimers or tetramers *in vitro*, with oligomerization-dependent activation of its GTP hydrolysis (26, 27). Specifically, GBP5 transitions from a nucleotide-free dimer to larger complexes upon GTP binding and then dissociates back into dimers following GTP hydrolysis (27). These conformational changes are critical to GBP5’s immune functionality (28). A unique feature of GBP5 is the presence of a CaaX motif at the C-terminus of the GED, essential for prenylation and membrane anchoring. This modification enables GBP5 to localize to intracellular membranes and interact with other proteins (3, 23). Prenylated GBP5 dimers predominantly associate with the Golgi apparatus and form granular structures within the host cell cytosol (29). This structural configuration supports its antiviral functions, such as reducing the infectivity of HIV-1 virions through its Golgi-localized prenylated form (8). Furthermore, GBP5 exhibits multiple splice variants, including hGBP5ta, which is specifically expressed in tumor tissues and demonstrates antigenicity, suggesting a potential role in tumorigenesis (30). These distinctive structural and functional attributes establish GBP5 as a key player in host defense and biological regulation, providing a foundation for its diverse roles in immune responses and disease pathogenesis.

## PHYSIOLOGICAL FUNCTIONS OF GBP5

Over the past several decades, interferons have emerged as critical regulators of both innate and adaptive immunity, influencing a wide range of pathological processes, including cancer, inflammation, and autoimmune diseases (31). As an IFN-inducible member of the GBP family, GBP5 plays a pivotal role in cellular autonomous immunity and is implicated in diverse physiological processes (1). This section explores GBP5’s multifaceted roles, focusing on its contributions to immune defense, cellular signaling, gene regulation, and pyroptosis.

### Immune defense and immune responses

As a member of the GBP family, GBP5 serves as a pivotal mediator of immune responses. It enhances innate immunity by promoting IFN production and pro-inflammatory signaling, fortifying host defenses (32). In *Branchiostoma japonicum*, elevated GBP5 expression in immune-related tissues highlights its role in host defense mechanisms (33). In viral infections, GBP5 expression increases significantly in A549 human lung epithelial cells infected with the influenza virus, where it augments virus-induced IFN production and suppresses viral replication. Conversely, GBP5 knockdown facilitates viral proliferation, underscoring its antiviral efficacy (34). GBP5 and GBP2 further bolster defenses against pathogens like HIV, measles virus, and Zika virus by inhibiting host protease furin (4, 35, 36). GBP5 also activates the NLRP3 inflammasome via a caspase-1-dependent pathway, inducing the production of pro-inflammatory cytokines such as IL-1β and IL-18 (37, 38). Its dimerization, which occurs in homo- and hetero-dimer forms, influences its subcellular localization and immune response modulation (29). Studies in mice reveal that GBP5 deletion increases susceptibility to various pathogens, emphasizing its critical role in mammalian immunity (39). Moreover, GBP5 is expressed in activated T cells, suggesting a role in regulating their proliferation, differentiation, and function (40). Collectively, GBP5 is integral to immune defense, and its diverse mechanisms offer promising avenues for infection control.

### Cellular signal transduction and regulation

GBP5 plays a central role in cell signaling, particularly during viral infections, partly through the activation of the NF-κB signaling pathway (32). It enhances IFN expression and related effectors while activating pro-inflammatory factors such as IL-6, IL-8, tumor necrosis factor-α, and NF-κB-dependent genes, thereby strengthening antiviral defenses (32). GBP5 also interacts with signaling proteins involved in NF-κB and interferon regulatory factor 3 activation, facilitating responses to pathogen-associated molecular patterns (32). Research has shown that GBP5 strengthens type I IFN expression and downstream effectors, such as 2′−5′ oligoadenylate synthetases, protein kinase R, and the human myxovirus resistance protein 1, reinforcing antiviral defenses (34). In extrapulmonary tuberculosis patients, GBP5 expression correlates with immune cell infiltration and diverse immune cell subtypes, suggesting a role in modulating immune cell functions (41). In intestinal epithelial cells, GBP5 inhibits HIV by activating toll-like receptor 3 signaling and is transferable via exosomes, enabling a broader antiviral capacity within tissues (42). These findings underscore GBP5’s regulatory influence on immune responses through intricate signaling networks.

### Gene regulation and inheritance

The GBP5 gene plays a significant role in genetic regulation and species-specific adaptations, particularly in disease resistance. Polymorphisms in the GBP5 gene locus are associated with outcomes of porcine reproductive and respiratory syndrome virus (PRRSV) infections, with certain mutations enhancing viral resistance (43–49). In Norduz goats, increased GBP5 copy numbers improve immune defenses, reflecting natural selection’s impact on genetic enhancement (50). Additionally, GBP5 has been identified as a candidate gene for feed efficiency in Swedish dairy cows, offering potential applications in livestock genetic improvement (51). Bioinformatic analyzes suggest that GBP5 may be regulated by microRNAs, indicating potential roles in autophagy, metabolism, and immune responses (52). Moreover, GBP5 exhibits transcriptional memory upon IFN-γ stimulation, revealing a unique regulatory feature in genetic control (53). Taken together, these findings position GBP5 as a critical player in genetic adaptation and immune system regulation across species.

### Pyroptosis

GBP5 is deeply involved in pyroptosis, a form of programmed cell death, and associated inflammatory processes. It can act as both a protective factor and an inducer of pyroptosis, notably activating the JAK2/STAT1 pathway in ovarian cancer cells, limiting growth and migration (54). In inflammatory conditions, GBP5 promotes chondrocyte pyroptosis by upregulating pyroptosis-related genes, such as NLRP3, Caspase-1, and Gasdermin D (GSDMD), through the NLRP3 inflammasome pathway, contributing to osteoarthritis progression (55). Studies also reveal that GBP5 can activate inflammasome complexes containing caspase-1 or caspase-4, initiating cell pyroptosis (56). During infection, GBP5 triggers GSDMD-mediated pyroptosis by facilitating caspase-11 activation, influencing pathogen invasion (57). In *Salmonella* infections, GBP5 enhances caspase-1 activation, increasing macrophage susceptibility to pyroptosis and aiding host defense (58). Bioinformatic studies also identify GBP5 as a pyroptosis-related gene enriched in NF-κB signaling pathways, suggesting its involvement in diseases like Sjögren’s syndrome (SS) (59). In summary, GBP5 plays a vital role in modulating pyroptosis and inflammation through pathways such as JAK2/STAT1 and NLRP3, offering insights into its functions in diverse pathological contexts.

## THE ROLE OF GBP-5 IN DISEASE

GBP5 plays a pivotal role in the pathogenesis of diverse diseases, including infections, cancers, immune disorders, and inflammatory conditions, where its expression levels exhibit dynamic changes and potential immunoregulatory functions (18, 55, 60, 61). In tuberculosis, GBP5 expression demonstrates variable patterns. Some studies report elevated GBP5 levels with strong diagnostic potential (60, 62, 63), while others identify downregulation in pulmonary tuberculosis patients. These discrepancies likely reflect distinct stages of infection, suggesting GBP5 as a promising biomarker for monitoring disease progression and therapeutic response (64). In colitis, GBP5 is closely linked to the composition and activity of the intestinal microbiota, emphasizing its critical role in maintaining gut immune balance. However, larger studies are needed to confirm this relationship (65). In oncology, GBP5 expression varies across cancer types. It is significantly upregulated in GC and oral squamous cell carcinoma (OSCC) (66, 67), but shows no notable changes in lung cancer, despite considerable alterations in its upstream transcriptional regulation. These findings may offer insights into the pathogenesis of lung cancer (68). GBP5’s antiviral activity has also garnered attention. It exhibits resistance to several viruses, including HIV-1, measles virus, and severe acute respiratory syndrome coronavirus 2 (SARS-CoV-2) (69). In Porcine reproductive and respiratory syndrome (PRRS), GBP5 expression fluctuates significantly, and specific polymorphisms are associated with increased abortion rates in sows, underscoring its critical role in antiviral immunity (45, 70). In mental health disorders, GBP5 exhibits distinct expression patterns. For instance, in cocaine use disorder (CUD), reduced GBP5 levels correlate with severe anhedonia, suggesting that GBP5 may contribute to this condition via immune and inflammatory dysregulation (71). Notably, GBP5 has also been identified as a gene with gender-specific effects. In osteonecrosis of the femoral head, elevated GBP5 expression in female patients may partly explain gender differences in disease prevalence and clinical presentations (72). In summary, GBP5 demonstrates diverse expression patterns and regulatory mechanisms across a wide range of diseases. Its involvement in infections, cancer, immune disorders, and other conditions highlights its potential as a diagnostic and therapeutic target. Further exploration of GBP5’s mechanisms and clinical implications may pave the way for innovative approaches to disease management.

### GBP5 in infection

GBP5 is markedly upregulated during infections, demonstrating diverse immunoregulatory functions in bacterial, viral, and parasitic infections. Its mechanisms and therapeutic potential vary significantly depending on the pathogen, with roles in cell pyroptosis, inflammatory regulation, and immune defense. A detailed summary of GBP5’s functions across various infectious diseases, including its mechanisms, species specificity, and clinical relevance, is presented in Table 1. These findings underscore GBP5’s broad involvement in infectious diseases and its promise as an immunomodulatory factor.

**TABLE 1:** A comprehensive overview of the role and mechanism of GBP5 in infectious diseases^*f*^

Diseases	Pathogens	Mechanism	Species	Potential value	Ref.
Bacterial infection					
Tuberculosis^*a*^	*Mycobacterium* ^*b*^	Activation of inflammasomes; Regulation of immune cell balance; Inhibition of macrophage apoptosis	Human and *M. mungi*	Early diagnosis, differential diagnosis, efficacy monitoring, and therapeutic targets of TB	(41, 60, 73–79)
Leprosy	*Mycobacterium leprae*	Induction of immune response	Human	Potential therapeutic targets	(80)
Brucellosis	*Brucella abortus*	Activation of inflammasomes; Promotion of pyroptosis	Mouse	Development of drugs or vaccines; Potential therapeutic targets	(57, 81, 82)
*Francisella novicida* infection	*Francisella novicida*	Promotion of IRGB10 recruitment to bacteria; AIM2 inflammasome activation	Mouse	Potential therapeutic targets	(38, 83)
*Salmonella enterica* infection	*Salmonella enterica*	Promotion of caspase-1 activation, SPI-1 expression, and enhanced pyroptosis	Mouse	Development of drugs or vaccines	(58)
*Burkholderia thailandensis* infection	*Burkholderia thailandensis*	Limitation of bacteria-induced cell fusion and intercellular spreading	Mouse	Potential therapeutic targets	(84)
*Stenotrophomonas maltophilia* infection	*Stenotrophomonas maltophilia*	Activation of inflammasomes involved in immune response	Mouse	Potential therapeutic targets	(85)
Sepsis^*c*^	*Escherichia coli*, Lipopolysaccharide (LPS)	Activation of NLRP3 inflammasome	Mouse	Candidates for anti-inflammatory therapy	(12, 86, 87)
Endotoxemia	*Escherichia coli*, LPS	Involvement in immune response	Mouse	Potential therapeutic targets; Diagnosis and treatment of diseases	(88, 89)
Viral infection					
HIV infection	HIV	Interference with the processing and integration of the HIV-1 Env protein	Human	Antiviral therapeutic targets; Development of novel antiviral strategies and approaches	(8, 24, 35, 90)
PRRS	PRRSV	Suppression of the downstream processes of PI3K	Pig	Development of vaccines and antiviral drugs; Genetic breeding and disease prevention/control; Development of immune enhancers	(44, 47–49, 70)
RSV infection	RSV	IFN-γ-induced GBP5 secretion of SH protein blocks replication	Human	Future research directions and clinical application potential	(91)
Influenza A virus infection	IAV	Enhanced expression of virus-induced interferons and related effectors; Interaction with the essential NF-κB regulatory complex to stimulate NF-κB signaling; Promotion of pro-inflammatory factor expression	Human	Potential targets for antiviral therapy and immune modulation	(32)
Herpes simplex virus type 2 Infection	HSV-2	*–^e^*	Human	Future research directions and clinical application potential	(92)
Corona Virus Disease 2019 (COVID-19)	SARS-CoV-2	–	Human	Potential diagnostic biomarkers	(93)
MNV infection	MNV	–	Mouse	Potential therapeutic targets	(94)
Bovine herpesvirus 1 infection	BoHV-1	–	Bovine	Disease prevention and control	(95)
Hepatitis B virus infection	HBV	Inhibition of viral replication; Interference with viral envelope gene processing	Human	Development of novel antiviral strategies and methods	(15)
Epstein-Barr virus infection	EBV	–	Human	Potential therapeutic targets	(96)
HAM/TSP	HTLV-I	Inhibition of viral replication	Human	Potential therapeutic targets	(97)
Multiple viral infections^*d*^	Various viruses	Reduction in viral glycoprotein assembly and obstruction of glycoprotein transport to the cell surface; Inhibition of viral dependency factor furin protease activity	Human	Future research directions and clinical application potential	(35, 98)
Parasite infection					
Toxoplasmosis	*T. gondii*	Activation of NADPH oxidase and NLRP3 inflammasome; Golgi localization, GTPase activity, and isoprenylation modification; Cooperation with GBP1 and GBP2	Human, mouse, and cat	Development of new diagnostic tools or therapeutic interventions	(99–103)
Leishmaniasis	*Leishmania*	Cooperation with other GBP family members	Mouse	Potential therapeutic targets	(104)

^*a*^ Tuberculosis (TB), including latent tuberculosis (LTBI), active tuberculosis (ATB), extrapulmonary tuberculosis (EPTB), severe tuberculosis, and tuberculous meningitis (TBM).

^*b*^ Mycobacterium, including *Mycobacterium tuberculosis* (Mtb) and *Mycobacterium mungi*.

^*c*^ Sepsis, including sepsis-associated liver injury (SALI).

^*d*^ Multiple viral infections, including HIV, MLV, SARS-CoV, SARS-CoV-2, and VSV, Zika virus, measles virus, and influenza A virus.

^*e*^ "–” indicates further studies are required to explore this aspect.

^*f*^ BoHV-1, Bovine herpesvirus 1; EBV, Epstein-Barr virus; HAM/TSP, human T cell lymphotropic virus-1-associated myelopathy/tropical spastic paraparesis; HBV, hepatitis B Virus; HIV, human immunodeficiency virus; HSV-2, herpes simplex virus type 2; HTLV-I, human T cell lymphotropic virus-1; IAV, influenza A virus; LPS, lipopolysaccharide; MNV, murine leukemia virus; PRRS, porcine respiratory and reproductive syndrome; PRRSV, porcine respiratory and reproductive syndrome virus; RSV, respiratory syncytial virus; SARS-CoV-2, severe acute respiratory syndrome coronavirus 2; T. gondii, Toxoplasma gondii.

### Bacterial infection

In bacterial infections, GBP5 plays a critical role in regulating inflammation, controlling cell death, and limiting pathogen spread. It activates inflammasomes, such as absent in melanoma 2 (AIM2) and NLRP3, to promote the release of IL-1β and IL-18, enhance pathogen clearance, and modulate inflammation, particularly in sepsis and endotoxemia (12, 38, 88). Examples include Mtb, *Brucella*, *Francisella novicida*, and *Stenotrophomonas maltophilia*, where GBP5 facilitates pathogen elimination and suggests potential for anti-inflammatory therapies (12, 38, 73, 81, 83, 85, 86, 105). GBP5 also induces pyroptosis to restrict pathogen survival. In *Mtb*, it inhibits caspase-3, preventing macrophage apoptosis and strengthening host defense (74). In *Salmonella enterica*, GBP5 activates caspase-1 and Salmonella pathogenicity island 1 (SPI-1) to induce pyroptosis, promoting intracellular pathogen clearance (58). Similarly, in *Brucella*, GBP5 disrupts the Brucella-containing vacuole, activates caspase-11 and GSDMD-mediated pyroptosis, and facilitates bacterial clearance (57). Additionally, GBP5 limits pathogen dissemination through non-traditional mechanisms. In *Burkholderia thailandensis*, GBP5 prevents bacteria-induced cell fusion, controlling local spread independently of inflammation or pyroptosis (84). Collectively, these findings highlight GBP5 as a potential biomarker for early diagnosis and a target for anti-infective therapies.

### Viral infection

GBP5 demonstrates multifaceted antiviral activity by inhibiting viral replication, modulating immune responses, and disrupting viral protein functions. It interferes with viral glycoprotein processes, including glycosylation, proteolytic processing, and transport, thereby exerting broad antiviral effects (69). Additionally, GBP5 can also control viral replication by modulating key molecules in antiviral innate immune signaling (18). In HIV infections, GBP5 inhibits replication by suppressing furin-mediated proteolytic processing of the viral gp160 Env precursor into functional gp120/gp41 subunits, compromising Env maturation and reducing virion infectivity, thereby establishing its potential as an anti-HIV target (8, 24, 35). In Human T cell lymphotropic virus-1 infections, GBP5 enhances interferon expression and restricts viral replication, particularly in Human T-cell lymphotropic virus-1-associated myelopathy/tropical spastic paraparesis (97). In HBV infections, GBP5 limits viral replication and envelope gene processing (15). In influenza A virus infections, it activates interferons, pro-inflammatory factors, and NF-κB signaling to enhance immunity (32). For PRRSV, GBP5 inhibits PI3K-mediated downstream processes, effectively suppressing viral entry and replication. Notably, genetic variations in GBP5 significantly influence swine resistance to PRRSV and correlate with production traits (e.g., weight gain), establishing its dual value for antiviral breeding and disease control strategies (49, 70, 106). In respiratory syncytial virus infection, IFN-γ-induced GBP5 impairs viral replication by promoting extracellular secretion of the small hydrophobic (SH) protein via direct interaction and Golgi apparatus-dependent vesicular trafficking, thereby depleting intracellular SH levels critical for viral propagation (91). In murine norovirus (MNV) infection, GBP5 upregulation during bacteriophage-induced antiviral responses correlates with suppressed viral replication, suggesting its potential immunomodulatory role in MNV control, though direct mechanistic evidence requires further validation (94). These findings highlight GBP5’s robust antiviral activity across species, providing insights for livestock breeding and antiviral drug development. However, further studies are necessary to elucidate its mechanisms and optimize clinical applications.

### Parasite infection

In parasitic infections, GBP5 exerts direct antiparasitic effects while enhancing host immunity. In *Toxoplasma gondii* infections, GBP5 is significantly upregulated in brain tissues, although mechanisms remain unclear (99). Evidence suggests that GBP5 activates nicotinamide adenine dinucleotide phosphate (NADPH) oxidase, stimulates the NLRP3 inflammasome, and enhances macrophage clearance of *T. gondii*. Localization to the Golgi apparatus via isoprenylation further restricts parasite survival (100–102). Additionally, GBP5 synergizes with other GBP family members, such as GBP1 and GBP2, to enhance host defense (102). In *Leishmania* infections, GBP5 collaborates with related proteins to boost immunity (104). Unlike its regulatory role in bacterial and viral infections, GBP5 exerts more direct antiparasitic effects, underscoring its therapeutic potential in parasitic diseases.

### GBP5 in cancer

The role of GBP5 in cancer has attracted increasing attention due to its involvement in tumor immune escape and regulation of the tumor microenvironment (61). Table 2 outlines GBP5’s mechanisms and significance across various tumor types. Despite variations in immune microenvironments and pathological characteristics among cancers, GBP5 consistently contributes to tumorigenesis and progression through its effects on tumor signaling pathways, immune microenvironment modulation, cell proliferation, migration, invasion, and pyroptosis. In GC, OSCC, hepatocellular carcinoma, breast cancer, and others, GBP5 activates pathways such as JAK1-STAT1/GBP5/CXCL8, IFN-γ/STAT1, TNF-α/NF-κB, and PI3K/Akt/mTOR, promoting tumor cell proliferation, invasion, programmed cell death ligand 1 (PD-L1) expression, and immune cell infiltration. These findings underscore GBP5’s dual role in facilitating cancer immune escape while simultaneously enhancing anti-tumor immune responses (9, 61, 107–110). In liver and ovarian cancers, GBP5 not only activates PI3K-AKT signaling but also modulates tumor metabolism and immune evasion via DNA methylation and miRNA networks (110, 111). In glioma, GBP5 drives tumor malignancy by activating the Src/ERK1/2/MMP3 pathway (112). Additionally, in cutaneous melanoma and ovarian cancer, GBP5 induces pyroptosis through the JAK2-STAT1-CASP1 axis, influencing cell death and immune responses (54, 113). In breast, lung, and pancreatic cancers, GBP5 promotes NLRP3 inflammasome assembly, contributing to immune regulation and potentially linking inflammation to tumor progression (114–116). In summary, GBP5 exhibits complex mechanisms in cancer, including regulation of pyroptosis, inflammasome activation, cell proliferation, invasion, immune evasion, and microenvironment remodeling. These insights emphasize GBP5’s potential as a diagnostic marker, therapeutic target, and prognostic indicator in cancer. Further research is needed to delineate GBP5’s regulatory pathways in diverse cancer types and to elucidate its evolving role within the tumor microenvironment, offering opportunities for advanced cancer immunotherapies and infection-related cancer interventions.

**TABLE 2:** A comprehensive overview of the role and mechanism of GBP5 in cancer

Diseases	Mechanism	Species	Potential value	Ref.
Gastric cancer^*a*^	Involvement in JAK1-STAT1/GBP5/CXCL8 signaling pathway, promoting gastric cancer cell proliferation and migration; participation in immune infiltration and transcriptional regulation; enhances immune defense and anti-tumor effects	Human	Diagnostic biomarker, potential therapeutic target, and disease prognosis evaluation	(9, 31, 117–119)
OSCC^*f*^	Promotes cell growth, influences cell cycle, enhances migration and invasion, and maintains cancer stem cell traits; activates IFN-γ/STAT1 and TNF-α/NF-κB pathways, influencing OSCC invasion and immune evasion; modulates tumor immune microenvironment by promoting immune cell infiltration and PD-L1 expression	Human	Diagnostic biomarker, potential therapeutic target, and disease prognosis evaluation	(61, 67, 107)
Breast cancer^*b*^	Activation of IFN-γ/STAT1 and TNF-α/NF-κB pathways promotes cancer cell invasion and metastasis; regulation of PI3K/Akt/mTOR axis and autophagy affects chemosensitivity; promotes NLRP3 inflammasome assembly; increases PD-L1 expression; modulates tumor immune microenvironment	Human	Diagnostic biomarker, potential therapeutic target, and disease prognosis evaluation	(108, 109, 115, 120)
Lung cancer^*c*^	Regulates tumor immune microenvironment; modulates immune cell function and activity; activates NLRP3 inflammasome; reprograms tumor-associated macrophages (TAM) into M1 phenotype through Photodynamic Therapy (PDT) for anti-tumor effects	Human and Mouse	Diagnostic biomarker, potential therapeutic target, and disease prognosis evaluation	(68, 114, 121, 122)
Ovarian cancer	Inhibits/promotes cancer cell proliferation, migration, and invasion; induces classical pyroptosis in ovarian cancer cells via JAK2-STAT1-CASP1 axis; involved in the pathways by which Bisphenol A (BPA) affects ovarian cancer progression; regulates miRNA and transcription factor networks in cancer cells	Human	Diagnostic biomarker, potential therapeutic target, and disease prognosis evaluation	(54, 111, 123, 124)
Hepatocellular carcinoma	Associated with immune cell infiltration; participates in PI3K-AKT pathway modulation, affecting cell migration and metabolism; promotes DNA methylation	Human	Disease prevention, diagnostic biomarker, potential therapeutic target, and disease prognosis evaluation	(110, 125, 126)
Skin cutaneous melanoma	Plays a role in pyroptosis	Human	Potential therapeutic target and disease prognosis evaluation	(30, 113, 127, 128)
Colon cancer	Modulates cancer immune microenvironment	Human	Potential therapeutic targets	(129)
Colorectal cancer	Inhibits cancer cell proliferation; promotes PD-L1 expression	Human	Potential therapeutic targets	(130)
Gastric adenocarcinoma	Modulates tumor immune microenvironment; enhances immune cell infiltration and activation	Human	Provides direction and ideas for further research	(66)
Gastrointestinal stromal tumors	–*^g^*	Human	Development of new immunotherapy strategies	(131)
Medullary carcinoma of the colon	Modulates the tumor immune microenvironment	Human	Prediction of immunotherapy response	(132)
Glioma^*d*^	Promotes cell proliferation, migration, and invasion via Src/ERK1/2/MMP3 axis; upregulates PD-L1 expression	Human and Mouse	Development of novel cancer treatment strategies; potential therapeutic target and disease prognosis evaluation	(14, 112, 133)
Uterine corpus endometrioid carcinoma	Regulates immune microenvironment in conjunction with CXCL9	Human	Potential therapeutic target and disease prognosis evaluation	(134)
Pancreatic adenocarcinoma	Functions within the tumor microenvironment; involved in pancreatic cancer immune response; promotes NLRP3 inflammasome assembly	Human	Potential therapeutic targets	(116)
bladder carcinoma	Enhances bladder response to BCG immunotherapy	Mouse	Potential therapeutic targets	(135)
Cutaneous T-cell lymphomas (CTCL)	GBP5 mutation leads to abnormal cell proliferation	Human	Potential therapeutic targets	(30)
Cancer^*e*^	Regulated by MLLT6, involved in tumor immune evasion and IFN-γ-associated immune resistance; promotes NLRP3 inflammasome activation	Human	Cancer immunosurveillance and potential therapeutic target	(136, 137)

^*a*^ Gastric cancer, including Epstein-Barr virus-associated gastric cancer (EBVaGC).

^*b*^ Breast cancer, including triple-negative breast cancer (TNBC).

^*c*^ Lung cancer, including non-small cell lung cancer (NSCLC) and small cell lung cancer (SCLC).

^*d*^ Glioma, including glioblastoma (GBM) and astroglioma (AG).

^*e*^ Cancer here denotes unspecified tumor types.

^*f*^ OSCC, oral squamous cell carcinoma.

^*g*^ "–” indicates further studies are required to explore this aspect.

### GBP5 in immune system diseases and inflammatory disorders

GBP5 plays a critical regulatory role in immune and inflammatory diseases by influencing diverse cell types, signaling pathways, and pathological mechanisms (Table 3). In disorders such as rheumatoid arthritis (RA), osteoarthritis (OA), phlebitis, and IBD, GBP5 activates the NLRP3 inflammasome, thereby increasing IL-1β and IL-18 production, which exacerbates local inflammation (55, 138–140). For instance, GBP5 expression is significantly elevated in peripheral blood mononuclear cells of patients with RA-associated interstitial lung disease, where it contributes to inflammatory responses and fibrosis through the activation of M1-like monocytes/macrophages (141). In OA, GBP5 regulates pyroptosis-related genes in chondrocytes via the IRF1/GBP5 axis, contributing to cartilage degradation (55). In IBD, GBP5 not only activates inflammasomes but also modulates gut microbiota composition, highlighting its dual role in regulating the microenvironment and driving disease progression (13). Beyond these diseases, GBP5 is increasingly recognized for its roles in other inflammatory conditions. In rosacea, GBP5 promotes M1 macrophage polarization through the NF-κB pathway, influencing cutaneous immune responses (142). In pulpitis, it balances pro-inflammatory cytokine levels to mitigate inflammation (7). In liver injury, GBP5 induces apoptosis via the NF-κB-inducing kinase (NIK) /NF-κB2 pathway, aggravating tissue damage (143). In inflammation-induced bone loss, GBP5 negatively regulates osteoclastogenesis, suggesting a protective role in bone health (144). Additionally, GBP5 is implicated in diseases such as SS and OA, where it directly promotes pyroptosis, emphasizing its role in cell death regulation and immune microenvironment stability (55, 59). In lupus nephritis (LN), GBP5 exacerbates immune-mediated renal damage by activating the NLRP3 inflammasome in renal tubular epithelial cells (145). In psoriasis-related renal damage, GBP5 activates the NF-κB/STAT3 pathway, driving inflammation and cell proliferation (146). Moreover, in type 1 diabetes, GBP5 may facilitate the recruitment and activation of inflammatory cells, though its specific mechanisms remain under investigation (147). Collectively, these findings underscore GBP5’s pivotal role in modulating inflammatory pathways, regulating cell death, and maintaining immune homeostasis. Its multifaceted actions in tissue damage control and immune regulation highlight its potential as both a biomarker and a therapeutic target for managing immune and inflammatory disorders.

**TABLE 3:** A comprehensive overview of the role and mechanism of GBP5 in immune system diseases and inflammatory disorders^*c*^

Diseases	Involved cell type	Mechanism	Cytokine stimulation	Species	Potential value	Ref.
RA	RASFs; RAW264.7 cells; monocytes	Attenuates cytokine-mediated inflammation and tissue damage; promotes the anti-inflammatory function of IFN-γ; activates the NLRP3 inflammasome and enhances IL-1β and IL-18 production; Activates M1-like monocytes/macrophages	rIL-1 β, TNF-α and IFN-γ	Human and mouse	Potential therapeutic targets	(138, 141, 148, 149)
Rosacea	Macrophages	Regulates M1 macrophage polarization via NF-κB signaling	LL­37	Human and mouse	Potential therapeutic targets	(142)
Psoriasis	HaCaT cells	Aggravates inflammation and cell proliferation through the NF-κB/STAT3 pathway	TNF-α	Human and mouse	Potential therapeutic targets	(146)
OA	Chondrocytes	Activates the NLRP3 inflammasome pathway through the IRF1/GBP5 axis, promoting pyroptosis-related gene expression in chondrocytes	TNF-α	Human and mouse	Potential therapeutic targets	(55)
SS	–^*d*^	Promotes pyroptosis; involved in immunoregulation	–	Human	Diagnostic biomarker and potential therapeutic target	(59)
Dental pulpitis	HDPSCs	Modulates inflammatory cytokine balance and inflammatory response	IFN-γ and LPS	Human	Potential therapeutic targets	(7)
IBD^*a*^	Colon mucosa of IBD patients; primary peritoneal macrophages; colon tissues and serums of the mice	Activates the NLRP3 inflammasome and enhances pro-inflammatory cytokine and chemokine production; modulates gut microbiota composition	IFN-γ, LPS, and DSS	Human and mouse	Diagnostic biomarker; Disease prevention and potential therapeutic targets	(13, 65, 140, 150–152)
LN	Human renal tubular epithelial cell; mouse cells in the renal cortex	Activates the NLRP3 inflammasome	LPS and ATP	Human and mouse	Potential therapeutic targets	(145)
Sarcoidosis	–	–	–	Human	Diagnostic biomarker and potential therapeutic target	(153)
Inflammation-induced bone loss	Primary osteoclasts	Negatively regulates osteoclastogenesis	LPS	Mouse	Potential therapeutic targets	(144)
Liver injury and inflammation	Primary hepatocytes; HEK293 and Hepa1-6 cells	Induces apoptosis via NIK/NF-κB2 signaling pathway	CCl_4_, LPS, TNF-α and IFN-γ	Human and mouse	Potential therapeutic targets	(143)
Behçet disease (BD)	Skin-infiltrating immune cells and monocytes in BD patients	–	–	Human	Potential therapeutic targets	(154)
Phlebitis^*b*^	BMDMs and PBMCs	Activates the NLRP3 inflammasome and enhances IL-1β production	LPS	Mouse	Potential therapeutic targets	(139)
Immune checkpoint inhibitors-associated myocarditis (ICIM)	–	Activates the NLRP3 inflammasome and drives IL-1β production	–	Human	Diagnostic biomarker and potential therapeutic target	(155)
Type 1 diabetes	–	Related to recruitment and activation of inflammatory cells	–	Human	Potential therapeutic targets	(147)

^*a*^ IBD, including crohn’s disease (CD) and ulcerative colitis (UC).

^*b*^ Phlebitis, including chemotherapy-induced phlebitis (CIP).

^*c*^ BD, Behçet disease; BMDMs, bone marrow-derived macrophages; DSS, dextran sulfate sodium; HaCaT, human immortalized keratinocyte; HDPSCs, human dental pulp stem cells; IBD, inﬂammatory bowel diseases; ICIM, immune checkpoint inhibitors-associated myocarditis; LN, lupus nephritis; LPS, lipopolysaccharide; OA, osteoarthritis; PBMCs, human peripheral blood mononuclear cells; RA, rheumatoid arthritis; RASFs, rheumatoid arthritis synovial fibroblasts; SS: Sjogren’s Syndrome.

^*d*^ "–” indicates further studies are required to explore this aspect.

### GBP5 in other diseases

GBP5 exerts diverse regulatory functions across various systemic diseases, primarily through mechanisms involving inflammasome activation, immune signaling modulation, and cell pyroptosis (Table 4). Its role in activating the NLRP3 inflammasome has been implicated in the pathogenesis of conditions such as pulmonary fibrosis (156), diabetic cardiomyopathy (157), asthma (158), radiation-induced brain injury (RIBI) (159), and preterm premature rupture of membranes (PPROM) (160). In PPROM, GBP5 is markedly upregulated in placental tissues, suggesting its contribution to preterm birth by mediating infection and inflammation. This highlights GBP5 as a novel factor in understanding preterm birth mechanisms (160). Furthermore, the GBP5/NF-κB/NLRP3 axis plays a critical role in conditions like fetal growth restriction and RIBI, emphasizing its central involvement in innate immunity and inflammatory regulation (159, 161). For example, GBP5 mRNA levels are significantly higher in South Asian type 2 diabetes patients of Dutch descent compared to those of European descent. This elevation correlates with IFN-γ levels, suggesting that GBP5 may amplify disease progression via IFN-γ-mediated inflammatory pathways. This finding positions GBP5 as a potential biomarker for monitoring inflammatory status and disease progression in diabetes (162). In neurological and psychiatric disorders, GBP5’s emerging roles are gaining attention. It has been proposed as a potential prognostic marker in cognitive decline (163). Additionally, in CUD patients with marked anhedonia, GBP5 expression is downregulated, linking it to the pathophysiology of anhedonia through immune and inflammatory dysregulation (71). This represents the first reported association between GBP5 expression and psychiatric symptoms, offering novel insights into the interplay between inflammation and mental health (71). In noneosinophilic chronic rhinosinusitis with nasal polyps (neCRSwNP), GBP5+ neutrophils, a specialized neutrophil subgroup, contribute to immune response regulation by expressing IFN-induced genes and PD-L1, playing a pivotal role in disease pathogenesis. This underscores GBP5’s extensive immunoregulatory capacity (164). GBP5’s roles in cardiovascular and bone diseases are equally noteworthy. In acute myocardial infarction, GBP5 has been identified as a potential therapeutic target due to its ability to modulate immune and inflammatory responses (165). In osteonecrosis of the femoral head, GBP5 expression correlates with disease progression, severity, and treatment outcomes, making it a promising marker for predicting disease risk and as a therapeutic target, particularly in relation to gender-specific factors (72). Furthermore, recent studies reveal that GBP5 promotes pyroptosis of Treg cells in hemorrhagic shock/resuscitation (HS/R) injury, underscoring its significant role in disease development (166). Overall, GBP5’s broad involvement in inflammation, metabolic signaling, disease progression, and cell pyroptosis highlights its potential applications in precision medicine. However, further research is essential to elucidate its disease-specific mechanisms across diverse populations, paving the way for refined mechanistic understanding and enhanced clinical translation.

**TABLE 4:** A comprehensive overview of the role and mechanism of GBP5 in other diseases^*b*^

Diseases	Mechanism	Species	Potential value	Ref.
Pulmonary fibrosis	Activates the NLRP3 inflammasome	Mouse	Potential therapeutic targets	(156)
HS/R	Influencing the survival and function of Treg cells	Rodent	Potential therapeutic targets	(166)
Nasal polyps	Regulates immune and inflammatory responses	Human	Potential therapeutic targets	(164)
RIBI	Promotes inflammasome assembly and activation through the GBP5/NF-κB/NLRP3 signaling axis; enhances the release of pro-inflammatory cytokines	Mouse	Potential therapeutic targets	(159)
Diabetic cardiomyopathy	Activates the NLRP3 inflammasome	Mouse	Potential therapeutic targets	(157)
COPD	–^*c*^	Human	Potential therapeutic targets	(167)
Asthma^*a*^	Activates the NLRP3 inflammasome	Human	Diagnostic biomarker and potential therapeutic target	(158, 168)
Celiac disease (CD)	–	Human	Diagnostic biomarker	(169)
Type 2 diabetes	Participates in IFN-γ-mediated inflammatory responses	Human	Evaluation of disease progression and prognosis	(162)
Post-traumatic complications	–	Mouse	Markers for early diagnosis	(170)
PPROM	Promotes the assembly of the NLRP3 inflammasome	Human	Potential diagnostic biomarker	(160)
Acute myocardial infarction (AMI)	Regulates immune and inflammatory responses	Human	Diagnostic biomarker and potential therapeutic target	(165)
FGR	Associated with innate immunity and the NF-κB pathway	Human	Disease intervention and potential therapeutic target	(161)
Cognitive decline	–	Mouse	Markers for disease prognosis	(163)
Atherosclerosis	–	Human and mouse	Novel biomarkers	(171)
Acute respiratory distress syndrome (ARDS)	Promotes pulmonary inflammation and interferes with the formation and function of autophagosomes.	Mouse	Potential therapeutic targets	(172)
CUD	–	Human	Diagnostic markers and evaluation of disease prognosis	(71)
Osteonecrosis	–	Human	Diagnostic biomarker and potential therapeutic target	(72)

^*a*^ Asthma, including eosinophilic asthma (EA) and neutrophilic asthma (NA).

^*b*^ AMI, acute myocardial infarction; ARDS, acute respiratory distress syndrome; CD, celiac disease; COPD, chronic obstructive pulmonary disease; CUD, cocaine use disorder; FGR, fetal growth restriction; HS/R, hemorrhagic shock/resuscitation; PPROM, preterm prelabor rupture of membranes; RIBI, radiation-induced brain injury.

^*c*^ "–” indicates further studies are required to explore this aspect.

## DISCUSSION

This review highlights the expression patterns and functional mechanisms of GBP5 across various diseases, emphasizing its multifaceted roles and the challenges associated with its clinical application. Notably, discrepancies in GBP5 expression across studies pose significant hurdles. For example, in tuberculosis, an infectious disease caused by Mtb, GBP5 expression varies markedly: some studies report significant upregulation in active pulmonary tuberculosis, supporting its potential as a diagnostic marker (60, 63), while others observe downregulation in similar patient populations (64). In latent tuberculosis infection (LTBI), GBP5 transcription is elevated but not statistically significant, suggesting its potential role in differentiating LTBI (75). Moreover, recent findings demonstrate that GBP5 effectively distinguishes active tuberculosis from other respiratory diseases, LTBI, and healthy controls (173). These inconsistencies may arise from variations in disease stage, immune background, and sample size, necessitating further exploration of GBP5’s dynamic expression across populations and pathological states. The dual role of GBP5 in disease progression is another critical consideration. Excessive activation or imbalanced expression of GBP5 can exacerbate disease severity. For instance, in IBD, GBP5 exerts a pronounced pro-inflammatory effect, with its overexpression correlating with disease onset (140). Suppression of GBP5 mitigates colitis symptoms (150), and GBP5-deficient mice exhibit altered gut microbiota, increasing probiotic populations and potentially preserving intestinal immune balance (13). Conversely, in CUD, GBP5 downregulation in patients with severe anhedonia indicates impaired immune and inflammatory regulation, contributing to psychiatric symptom exacerbation (71). These findings underscore the importance of tailored therapeutic approaches targeting GBP5, accounting for disease stage and patient-specific conditions to avoid adverse effects. Despite its diagnostic and therapeutic potential, limitations in GBP5 research remain. Ethnic and genetic variability significantly influence its expression. For example, South Asian type 2 diabetes patients of Dutch descent exhibit higher GBP5 mRNA levels than European-origin patients, correlating with IFN-γ protein levels (162). Similarly, in femoral head necrosis, GBP5 expression is higher in female patients and correlates with disease progression and severity (72). These differences highlight the need for studies involving larger and more diverse cohorts to evaluate GBP5’s generalizability as a therapeutic target.

The therapeutic potential of GBP5 spans a wide range of diseases. For example, synthetic steroid 5α-Androst-3β has been shown to inhibit the GBP5/NLRP3 axis, reducing microglial activation and pro-inflammatory cytokine release, thereby protecting neurons in neurological disorders (159). The Exportin 1 (XPO1) inhibitor Selinexor similarly targets GBP5 to suppress the NLRP3 pathway, alleviating pulmonary fibrosis (156). In colitis, histone deacetylase 3 (HDAC3) regulates the GBP5-NLRP3 axis in macrophages, alleviating inflammation and disease severity (150). Other compounds, such as Aescin and Sinomenine (SIN), modulate GBP5 to mitigate symptoms in chemotherapy-induced phlebitis and rheumatoid arthritis, respectively (139, 148). GBP5 also influences cell pyroptosis and immune responses. In the context of diabetic cardiomyopathy, bone morphogenetic protein-7 (BMP-7) alleviated inflammation-induced pyroptosis by inhibiting the TLR4-NLRP3 complex, which is activated by GBP5 and NIMA-related kinase 7 (Nek7). This highlights a unique regulatory mechanism by which GBP5 controls inflammatory cell death (157); loganic acid, a compound extracted from traditional Chinese medicinal compounds, is capable of inhibiting Treg cell pyroptosis mediated by GBP5, offering a novel strategy for the treatment of hemorrhagic shock and reperfusion injury (166). In antiviral and anti-tumor immunity, GBP5 shows diverse roles. In lung cancer, photodynamic therapy, namely a novel approach that integrates the photodynamic effect with anti-tumor immunotherapy, induces GBP5 expression in macrophages, promoting their polarization toward anti-tumor activity (121). In HSV-2 infection, the synthetic DNA analog poly (dA:dT) activates the Retinoic Acid-inducible Gene-I (RIG-I) signaling pathway, enhancing GBP5 expression for antiviral protection (92). These findings suggest that GBP5 can be specifically targeted to modulate immune responses in both viral and tumor-related diseases. Additionally, in respiratory diseases such as COPD, the RNA-binding protein Alpha-2-Glycoprotein 1 (AZGP1) downregulates GBP5, modulating epithelial hyperplasia during disease progression (167).

Given GBP5’s complex mechanisms and broad therapeutic implications, future research should prioritize: (i) Dynamic Profiling: Investigating GBP5 expression across varying immune backgrounds, disease stages, and conditions such as tuberculosis and IBD to refine its role as a biomarker. (ii) Individual Variability: Examining GBP5 expression in relation to ethnicity, gender, and genetic factors to enable personalized therapeutic strategies. (iii) Targeted Therapies: Developing precision interventions for infections, tumors, immune, and inflammatory diseases by modulating GBP5 activity. (iv) Safety Assessment: Evaluating the dual role and safety of GBP5 to minimize risks associated with its overactivation or imbalance in clinical applications. (v) Clinical Validation: Conducting large-scale, multi-center clinical trials to confirm GBP5’s diagnostic and therapeutic utility across diverse patient populations and disease contexts.

In summary, GBP5 represents a promising therapeutic target with potential for advancing disease prevention, diagnosis, and treatment. However, its successful clinical translation depends on overcoming current knowledge gaps and addressing its variability across populations and disease states.

## CONCLUSIONS

This review provides a comprehensive analysis of the diverse functions of GBP5 in various diseases, highlighting its potential as a biomarker and therapeutic target in infection, tumorigenesis, and immune regulation. However, significant variations in GBP5 expression and function across disease contexts and populations—driven by immune status, genetic background, and disease stage—pose challenges to its clinical reliability and applicability. Future research on the dynamic regulatory mechanisms of GBP5 and its role in various diseases will be essential for advancing its clinical applications and therapeutic potential.
